# Extirpation of an Abdominal Tumor of Large Size

**Published:** 1847-08

**Authors:** Daniel Brainard

**Affiliations:** Professor of Surgery in Rush Medical College, Attending Surgeon of the Chicago Hospital, &c.


ILLINOIS AND INDIANA

MEDICAL AND SURGICAL JOURNAL.

Vol. II NEW SERIES.—AUGUST, 1847. No. 3.

### PART I.—ORIGINAL COMMUNICATIONS.

ARTICLE I.

*Extirpation of an Abdominal Tumor of large size.* By DANIEL BRAINARD, M.D., Professor of Surgery in Rush Medical College, Attending Surgeon of the Chicago Hospital, &c.

John Meaten, Æt. 38 years, an Englishman, gardener, entered the hospital, April 5th, 1847, on account of a secondary venereal affection of the skin and a suppurating gland in the groin. His constitution had been greatly impaired by disease and remedies employed against it, although his general health was tolerably good. In addition to the syphilitic affection, he labored under a complication of surgical diseases which rendered his case one of much interest. He had an ancient unreduced dislocation of the left os humeri upon the dorsum of the scapula, and a large tumor of the abdomen. He was treated first for the bubo and constitutional disease; and these having been removed, attention was more particularly directed to the abdominal tumor.

It presented the following appearances: resting upon the right iliac region, it ascended to the right hypochondriac, encroached upon the epigastric, occupied the whole of the umbilical, and terminated upon the edge of the left lumbar region. It projected considerably, and appeared nearly as large as the head of an adult. Its surface was hard, irregular, and insensible. It appeared to be placed directly against the abdominal wall, was very movable, and could be perfectly circumscribed on every side. It could be pressed upward so as in a great measure to be buried beneath the false ribs to the left side so as to distend the parietes there, and by embracing it upon both sides it could, in a good measure, be lifted from its situation.

Its history, as far as observed by the patient, was as follows: About six months previously, he first perceived it situated midway between the umbilicus and the crest of the right ilium. It was of the size of an orange, hard, insensible, and very movable. Since that time it has regularly increased in size, rapidly of late—fully one-third since his residence in the hospital.

*Diagnosis.*—In regard to its character and situation, we expressed the opinion that it was fibrous in its texture, and located in the omentum. Its fibrous nature was inferred from its great hardness; its origin and situation were arrived at by the method of exclusion. Thus, it was not difficult to ascertain that it resulted neither from the pelvis, kidney, liver, pancreas, or stomach, from its situation when first observed. It was as easy to infer, with great certainty, that it was not situated in the mesentery or closely connected with the digestive organs; as in either of these cases, it would have shown its effect upon the system by deranging the nutritive action. In short, a careful examination of the case and a consideration of the distinctive signs of the different tumors of the abdomen, and of the diseases of the organs situated in that cavity, were sufficient, as is usually the case, to indicate, with great certainty, the seat and probable nature of the disease.

*Treatment.*—In reference to this, there was room for more difference of opinion. Should it be left to itself? Should it be removed by operation? These were the alternatives; for medical treatment promised nothing, nor did any surgical means, short of its removal, give better hopes. Its rapid growth preceded the hope of its remaining stationary for a length of time. It must soon encroach upon the viscera and interfere with their action; and it was important that a decision should be arrived at before adhesions and its size should preclude the possibility of relief. It was the patient himself who first seriously entertained thoughts of its extirpation. He was possessed of great firmness, with intelligence and a cheerful temper. After being told that an operation was practicable, although dangerous, he was not urged to or from its adoption; but, after some days reflection, he said that he wished to be operated upon, and that he had selected Monday, May 12th, for its performance.

*Operation.*—This was accordingly done on the morning of the day, with the assistance of Drs. Maxwell, Blaney, and Huber, and in presence of several physicians and the medical class in attendance upon the Hospital, as follows. The patient was placed upon a table, with the head raised, and was allowed to take a few inhalations of the ethereal vapor, which produced spasmodic action of the muscles of the back which continued for about a minute. (He had tried the inhalation the day before with the happiest effect.) These having subsided, and the tumor being pressed forward by assistants, an incision was made in the linea alba six inches before the umbilicus. The abdominal wall being divided, the omentum was brought into view. and on separating this in the interval of its vessels, the morbid mass exposed. Passing the finger around it, no adhesions were found, and where it was fully circumscribed above, the hands of the assistants still forcing it forwards, the patient made an effort and expelled it suddenly from the abdomen. It was immediately perceived that the pedicle of the tumor was composed of omentum, the vessels of which were thus greatly enlarged. A ligature was passed around it, and the tumor removed; but this was found insufficient, and the arteries to the number of eight were tied separately. The ligatures were cut close, the wound carefully cleansed, and its sides brought together and retained by stitches of interrupted suture and adhesive straps, and a bandage pinned around the body. About eight ounces of blood were lost during the operation, mostly from the first incisions. Including the dressing, the operation lasted about twenty minutes, and was well borne by the patient, who was little depressed by it, talking cheerfully until it was finished. He was placed in his bed, and a full dose of opium administered.

*Evening.*—No pain. Pulse 100. Feels well.

MAY 13th—*Morning.*—Has had hœmorrhage, apparently to the extent of six or eight ounces, during the night. Respiration frequent, with much mucous and sibilant rattle. Pulse 120, weak. Applied a sinapism over the chest; gave mucilaginous drink, with wine and nitre.

MAY 14th.—Feels much better. Pulse 100. Mucus rattle abundant. Little pain or tension of abdomen. Urine discharged regularly and of natural appearance. Has slept well. Continued counter irritation with a decoction of Senega as expectorant. Diet of soup and gruel, with toast water for drink.

MAY 15th.—Some reaction. Abdomen fuller and tender. Respiration hurried. Pulse 110. Has slept well. Urine natural. Gave a saline purge, with enemata of ol. ricini and ol. terebinth. aa ℥i with mucilage. Towards evening had stools which relieved the tension.

MAY 16th.—Has rested well, and taken soup with some relish. Pulse 110, weak. Respiration frequent. Mucous in bronchial tubes accumulating. Abdomen full but soft, and not very tender. Counter irritation and stimulating expectorants continued.

*Evening.*—Is evidently sinking from accumulation of mucus in the air passages. Wine, with capsicum and external warmth, was made use of. He died on the morning of the 17th; being five days after the operation.

*Examination ten hours after Death.*—Abdomen little more tumefied than in its natural state. Wound adhered throughout nearly its entire extent. The small intestines near the wound, adhered to the wall and to each other by recently effused lymph. Other parts of the peritoneum presented a healthy appearance. Small intestines, moderately distended with gas, and exhibited considerable vascularity of their coats.

The veins around which ligatures had been placed in connection with the arteries, were of large size, and filled with coagulated blood, but presented internally no trace of inflammation. No hœmorrhage had taken place from the ligated vessels. Stomach and kidneys healthy. Liver somewhat larger than natural, of a pale yellow color, and fatty feel. The pancreas was dry, firm, and indurated. No morbid growths or other diseased appearances were formed in the abdomen.

The cavities of the right side of the heart were distended with fibrinous concretions of extreme tenacity. The air cells were distended with serum, which flowed freely when they were cut. The right lung was extensively adherent by its pleura to the thoracic wall, and at the line of junction of the upper and lower lobes there was a spot about two inches in diameter, of a dark brown color, and entirely destitute of tenacity, being incapable of resisting the slightest touch. Both lungs were crepitant throughout.

The muscles of the entire system were firm, and in many places partially transformed to fibrous tissue. The tendons were thickened, rough, and adherent; and to the ligaments of the articulations at several points, were found indurated fibrous masses sufficient to impede materially the movements of the joints. In all the articulations of the hands and feet were found abundant chalky deposits. These affections of the joints, and fibrous and muscular tissues were doubtless the result of the ancient venereal affection.

*Examination of the Tumor.*—This, in size and form, strikingly resembled the heart of an ox. The base having been directed upward and to the right side. It was hard, irregular, and enveloped in part by omentum. On laying it open it creaked under the knife, was of a light gray color, very vascular at some points, and cartilaginous and bony at others. Along the back part extended an ossified piece, two inches in length, and branched in various directions.

Weight, three pounds ten ounches; length, nine inches; breadth, six inches; circumference, twenty-one inches.

*Remarks.*—In this case the cause of death seemed to be bronchitis, and the consequent accumulation of serum and mucus in the air cells and tubes. So far as the shock of the operation was concerned, the hœmorrhage, the subsequent reaction and inflammation, with the appearances on dissection, there is nothing to warrant the belief that death would have ensued independently of this complication.

The state of the constitution, enfeebled by disease and medicine was not the most favorable for resisting the effects of severe injury.

